# Meta-epidemiological assessment and evaluation of certainty of evidence in systematic reviews with meta-analyses of cognitive behavioral therapy and psychodynamic/psychoanalytic psychotherapy: an umbrella review protocol

**DOI:** 10.3389/fpsyt.2025.1664393

**Published:** 2025-10-01

**Authors:** Rogério Lerner, Bárbara Gonçalves, Caroline Martins Dias, Júlia Benvenutti Gerotto, Tayane Aparecida Teizen Rufino, Yasmin Meireles Aragão, Izabella Lopes de Arantes

**Affiliations:** ^1^ University of São Paulo, São Paulo, Brazil; ^2^ Mackenzie Presbyterian University, São Paulo, Brazil

**Keywords:** AMSTAR-2, quality, systematic reviews, grade, meta-epidemiology, umbrella review

## Abstract

**Background:**

Psychodynamic/psychoanalytic psychotherapy (PPT) and cognitive behavioral therapy (CBT) are two major therapeutic modalities widely used in clinical practice and supported by distinct theoretical and empirical bases. Persistent debate–particularly around PPT’s evidentiary strength–underscore the need to critically appraise the methodological quality and certainty of evidence of systematic reviews with meta-analyses (SRMAs) that underpin claims regarding its effectiveness, particularly in comparison with CBT.

**Objective:**

This umbrella review will assess and compare the methodological rigor (AMSTAR-2) and certainty of evidence (GRADE) of systematic reviews with meta-analyses (SRMAs) of randomized controlled trials (RCTs) of CBT and PPT, published between 2015 and 2024.

**Methods:**

We will search Embase, The Cochrane Library, PsycINFO, PubMed, and Web of Science for peer-reviewed SRMAs of RCTs of CBT and PPT across various mental disorders. Methodological quality will be assessed using AMSTAR-2, and their certainty of evidence will be evaluated using the GRADE system. Descriptive, correlational, and comparative analyses will examine associations between methodological quality, certainty of evidence, and reported SRMA-level effect patterns, and be used to synthesize the findings. Sensitivity analyses will address quality threshold, comparator class, psychiatric diagnosis, and quality thresholds; multiple testing will be corrected by Holm-Bonferroni.

**Discussion:**

By evaluating and comparing the volume, methodological rigor, and certainty of evidence of SRMAs CBT and PPT, this umbrella review aims to contribute to the ongoing debate on evidence-based psychotherapy, inform clinical decision-making, guide future research, and support evidence-informed public health policy.

**Systematic Review Registration:**

PROSPERO (CRD420250619644).

## 1 Introduction

For several years, psychoanalysis and its variant, psychodynamic/psychoanalytic psychotherapy (PPT), have been debated among clinicians, especially in comparison to cognitive behavioral therapy (CBT). This debate is largely due to differences in theoretical foundations and methodological approaches to treatment outcomes (1, 2).

PPT evolved from Freud’s psychoanalytic theory and focuses on exploring unconscious processes, past experiences, and unresolved conflicts that influence present behavior (3). PPT emphasizes the therapeutic relationship, transference interpretations, and exploration of early developmental experiences that shape current functioning. Advocates of PPT argue that this approach offers deeper, more lasting solutions to psychological issues. Critics, however, often point to the lack of large-scale empirical studies—specifically, randomized controlled trials (RCTs)—that provide rigorous evidence of its effectiveness. The often longer-term nature of PPT poses challenges for empirical research, as it is difficult to measure outcomes in the same way as with shorter therapies (4).

In contrast, CBT is based on the premise that psychological distress is often maintained by dysfunctional patterns of thinking and behavior. Thus, CBT focuses on modifying dysfunctional thinking and behavior patterns through structured, time-limited (brief-term) interventions. CBT has gained widespread recognition for effectively treating psychological disorders such as depression, anxiety, and post-traumatic stress disorder. However, some critics argue that, although CBT can rapidly alleviate symptoms, it may not address underlying emotional issues affecting long-term psychological health (5–8).

The debate over the comparative effectiveness of CBT and psychodynamic psychotherapy (PPT) has gained global relevance, influencing clinical guidelines, public policy, and psychotherapy research worldwide. For instance, the National Institute for Health and Care Excellence (NICE) recommends CBT and behavioral activation as primary treatments for depression; short-term psychodynamic psychotherapy (STPP) is considered a secondary option due to its cost-effectiveness. Similarly, the Royal Australian and New Zealand College of Psychiatrists’ (RANZCP) guidelines prioritize CBT. The latter discusses STPP separately, reflecting regional nuances in evidence-based practices (9). Furthermore, meta-analyses suggest that PPT could be as effective as CBT for certain disorders, though the longevity of its effects remains uncertain (6, 10).

Discussions about adapting CBT protocols and maintaining the relevance of PPT in diverse sociocultural contexts show that this debate transcends geographical and systemic boundaries, influencing clinical practices and health policy formulation. In recent years, several reviews have examined the effectiveness of these two approaches, yielding distinct yet simultaneous results (5–8). However, findings remain fragmented. Most systematic reviews analyze CBT and PPT separately and apply heterogeneous inclusion criteria, comparators, and outcomes. These reviews often reach conflicting conclusions (11–13).

Additionally, a proliferation of systematic reviews has been documented, particularly those on CBT. For instance, Fordham et al. (14) identified 494 CBT systematic reviews covering various conditions and populations, highlighting the wealth of available secondary evidence. Despite this volume, the methodological quality of these reviews varies considerably. Recent assessments (15, 16) indicate that 40% to 70% of these reviews are classified as low or critically low according to tools such as A MeaSurement Tool to Assess systematic Reviews 2 (AMSTAR-2) (17).

In this context, an umbrella review is a suitable method for critically evaluating the quality of systematic reviews, particularly when it is limited to reviews that include meta-analyses, as it allows for the integration of diverse findings into a comprehensive synthesis. This approach follows consolidated guidelines, such as those proposed by the Joanna Briggs Institute, using validated instruments, such as AMSTAR-2, to assess methodological rigor and risk -of- bias. Furthermore, understanding the relationship between the methodological quality of reviews and the reported effect sizes is essential to properly interpreting the evidence base (18, 19).

Methodological research shows that low-quality reviews often overestimate the benefits or underestimate the risks of interventions due to biases such as selective reporting, incomplete literature searches, and inadequate inclusion criteria. For instance, adjusting for publication bias can lower effect estimates by an average of 15.5%, potentially altering the statistical significance and clinical relevance of the results (20).

Exploring this association increases the reliability of conclusions and provides a more solid basis for evidence-based clinical guidelines and public policies (21). Thus, the aim of this protocol is to critically assess review-level methodological quality and certainty of evidence of systematic reviews with meta-analyses (SRMAs) examining the effectiveness of PPT and CBT in treating mental disorders. We will also investigate the association between methodological quality and reported effect sizes. The result will be a comprehensive, high-level synthesis of the available evidence.

### 1.1 Objectives

#### 1.1.1 Main objective

To assess and compare the methodological rigor and certainty of evidence of SRMAs of RCTs evaluating the efficacy of CBT and PPT.

#### 1.1.2 Specific objectives

1) to quantify SRMAs of RCTs on CBT and PPT; 2) to categorize SRMAs on CBT and PPT by their methodological quality; 3) to categorize SRMAs on CBT and PPT by their certainty of evidence; 4) to examine correlations between methodological quality of the included reviews and effect sizes through concordance; and 5) to report average AMSTAR-2 ratings of SRMAs of CBT and PPT addressing different psychopathologies (i.e., depression, anxiety, posttraumatic stress disorder, borderline personality disorder, obsessive-compulsive disorder, eating disorders, somatic symptom disorder, attention deficit hyperactivity disorder, substance use, schizophrenia, chronic mental disorder, substance-related disorders, dissociative disorder, and bipolar disorder).

## 2 Methods

This umbrella review will evaluate SRMAs of RCTs on CBT and PPT in treating several mental disorders to improve access to information regarding mental health treatment options. Additionally, we will explore possible comparative and correlation associations between the review-level methodological quality and certainty of evidence.

**Figure 1**.

**Figure 1:**
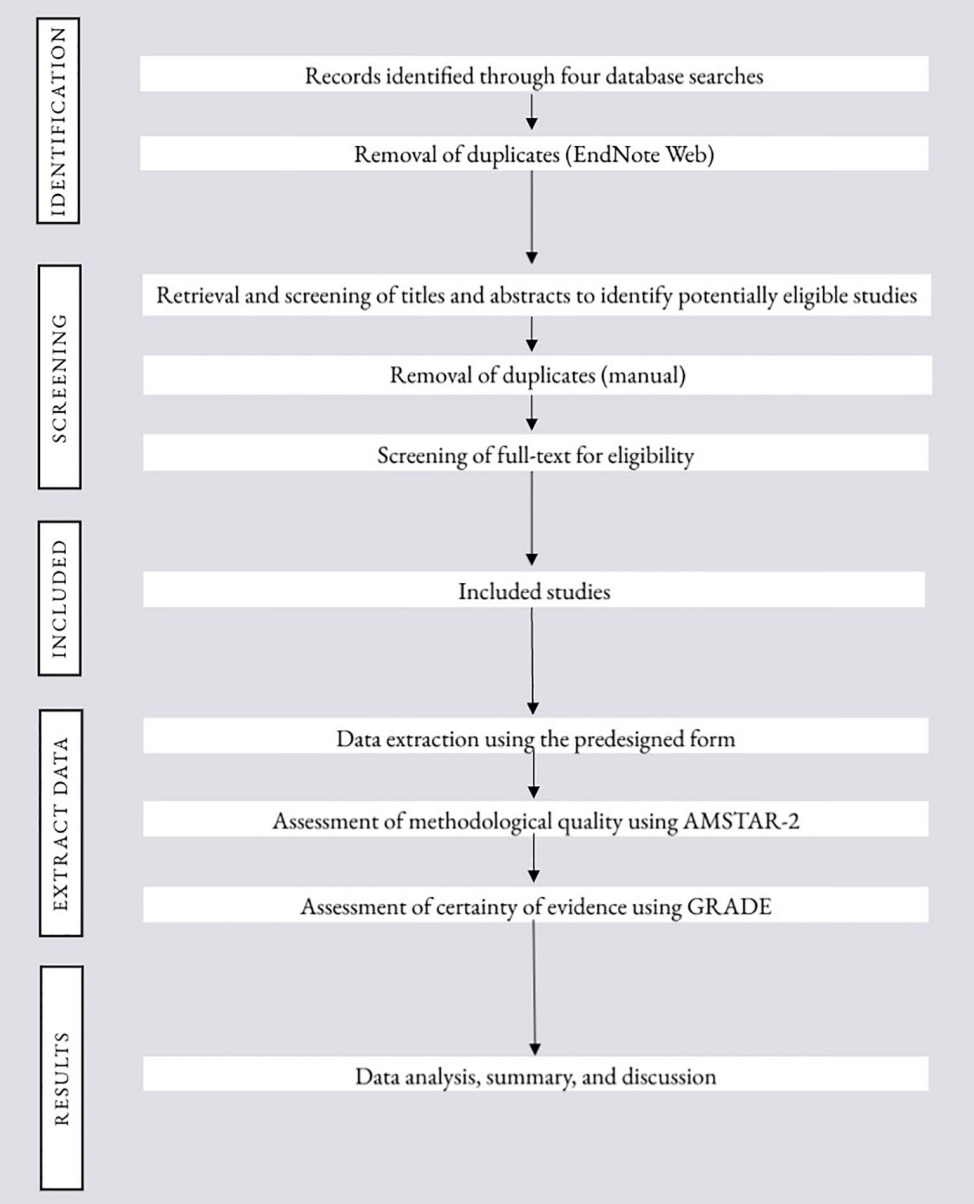
Flowchart of the study procedure.

### 2.1 Study protocol and registration

This protocol is registered in the International Prospective Register of Systematic Reviews (PROSPERO) database under registration number CRD420250619644 and follows the Preferred Reporting Items for Systematic Reviews and Meta-Analyses (PRISMA 2020) for reporting (22) and Preferred Reporting Items for Overviews of Reviews (PRIOR) for overviews (23). Any amendments to the protocol will be documented and reported.

### 2.2 Eligibility criteria

#### 2.2.1 Inclusion criteria

Inclusion criteria were defined according to the PICOS strategy (P = participant, I = intervention, C = comparator, O = outcome, S = study design). Only full-text systematic reviews published in peer-reviewed journals will be considered for inclusion. Restricting meta-epidemiological appraisal to SRMAs aligns with evidence hierarchies that place systematic reviews/meta-analyses at the top and ensures we assess the very synthesis agencies and governments use as the main instrument for policies. Regarding the time window rationale, **Table 1**.

**Table 1:** Umbrella review eligibility criteria.

Inclusion criteria	
Participants	SRMAs of RCTs on CBT and/or PPT involving participants with depressive disorders, anxiety disorders, trauma- and stressor-related disorders, dissociative disorders, obsessive-compulsive disorder, eating disorders, somatic symptom disorders, attention-deficit/hyperactivity disorder, substance related disorders, personality disorders, bipolar disorder, or schizophrenia spectrum disorders. There are no restrictions on age, sex, gender, or type of intervention (individual or group, brief or long-term).
Intervention	In-person and/or Internet-delivered psychotherapy using CBT and/or PPT as the main interventions will be considered.
Comparator	As long as CBT or PPT are the primary approaches, reviews may compare CBT or PPT with each other or with any of the following: other psychotherapeutic approaches, pharmacological interventions, waitlist, no treatment, treatment as usual (TAU), pill or psychological placebo, or psychoeducation.
Outcome	SRMA-level treatment efficacy or effectiveness at acute post-treatment (mental distress symptom reduction and/or improvement in quality of life), methodological quality (AMSTAR-2), and certainty of evidence (GRADE).
Time frame	SRMAs published between January 1, 2015 and December 31, 2024; English, Portuguese, or Spanish.
Study type	Peer-reviewed SRMAs that include only RCTs.
Context	SRMAs of RCTs on CBT and/or PPT conducted in a face-to-face or online context, both outpatient and inpatient treatment.

#### 2.2.2 Exclusion criteria

(i) Systematic reviews that do not assess treatment efficacy or effectiveness; (ii) systematic reviews without meta-analyses; (iii) SRMAs that do not focus on CBT or PPT; (iv) SRMAs that include non-randomized designs; (v) SRMAs of RCTs of interventions using solely artificial intelligence interventions and/or mobile health tools; (vi) SRMAs published in languages other than English, Portuguese, or Spanish (language restriction will be acknowledged as a limitation); and (vii) book chapters, dissertation, and thesis.

### 2.3 Search strategy

Searches will be conducted in The Cochrane Library, PsycINFO, PubMed, Embase, and Web of Science using validated systematic reviews filters (e.g., ISSG), terms for meta-analysis, and controlled vocabulary (MeSH where applicable). **Tables 2** and **3** present PubMed-specific strings. Equivalent strategies were translated and adapted for each database (e.g., controlled vocabulary and operators) to ensure functional equivalence. We will restrict inclusion to articles published from 2015 onward to ensure comparability under current evidence-based synthesis standards and contemporary psychotherapy delivery. From 2015, core guidance became available and widely adopted, for example, PRISMA for protocol reporting (22), and AMSTAR-2 for review quality. Reviews before this timeframe might not have pre-registered protocols or used these tools, which would systematically disadvantage older reviews for historical–and not methodological–reasons, and compromise the present study. This time window also captures the maturation and scale-up of Internet-delivered psychotherapy and updated diagnostic, as well as clinical frameworks (e.g., post-DSM-5), aligning included evidence with contemporary practice and outcomes.

**Table 2:** PubMed search strategy for PPT studies.

(“psychodynamic therapy”[All Fields] OR “dynamic therapy”[All Fields] OR “PPT”[All Fields] OR “psychoanalytic therapy”[All Fields] OR “psychodynamic psychotherapy”[All Fields] OR “dynamic psychotherapy”[All Fields] OR “psychoanalytic psychotherapy”[All Fields] OR “psychotherapy, psychodynamic”[Mesh] OR “psychoanalytic therapy”[Mesh] OR psychodynamic*[tiab] OR psychoanalytic*[tiab] OR “dynamic psychotherap*”[tiab] OR “psychodynamic psychotherap*”[tiab] OR “psychoanalytic psychotherap*”[tiab]) AND (systematic[sb] OR “systematic review”[tiab] OR “systematic reviews”[tiab]) AND (meta-analysis[pt] OR “Meta-Analysis as Topic”[Mesh] OR “network meta-analysis”[Mesh] OR “network meta-analyses [tiab] OR meta analy*[tiab] OR metaanaly*[tiab] OR “meta-regression”[tiab] OR metaregression[tiab]) AND (random*[tiab] OR “Randomized Controlled Trials as Topic”[Mesh])

**Table 3:** PubMed search strategy for CBT studies.

(“cognitive behavioral therapy”[All Fields] OR “cognitive behavioural therapy”[All Fields] OR “CBT”[All Fields]) AND (“Cognitive Behavioral Therapy”[Mesh] OR “Cognitive Therapy”[Mesh] OR cbt[tiab] OR “cognitive behav* therap*”[tiab] OR “cognitive therap*”[tiab]) AND (systematic[sb] OR “systematic review”[tiab] OR “systematic reviews”[tiab]) AND (meta-analysis[pt] OR “Meta-Analysis as Topic”[Mesh] OR “network meta-analysis”[Mesh] OR meta analy*[tiab] OR metaanaly*[tiab] OR “meta-regression”[tiab] OR metaregression[tiab]) AND (random*[tiab] OR “Randomized Controlled Trials as Topic”[Mesh])

### 2.4 Study selection

Search results retrieved from the five databases will be exported to EndNote Web for sorting and automatic de-duplication, then exported to Rayyan (24) for manual de-duplication and blinded screening. Titles and abstracts of remaining studies will be screened for a first assessment against eligibility criteria, followed by full-texts evaluation.

The first search results will be equally divided among four groups, each consisting of two independent reviewers. For each stage of the selection process, both reviewers in each group will independently screen the studies and have their work checked by a third reviewer. Inter-rater agreement will be reported with Cohen’s κ. Any disagreement between reviewers will be resolved by a third reviewer and, if necessary, by discussion between the reviewers and a senior independent reviewer (RL), ensuring consistency in reviewer pairs throughout the process. The pair of reviewers will remain consistent throughout the duration of the study (JBG and CMD, BG and BAV, TTR and YMA, and ILA and ARS). A PRISMA-compliant flowchart will be used in the report to illustrate the process of study selection.

### 2.5 Data extraction

For each therapeutic approach, the same pairs of reviewers who conducted study selection will independently extract review-level data using a predesigned form based on the JBI Data Extraction Form for Review for Systematic Reviews and Research Syntheses, as well as AMSTAR-2 and GRADE templates. As for the predesigned data form, we will retrieve the following review-level information:

- Bibliographic features: authors’ names and countries, year of publication, language of publication, journal, protocol registration, and funding sources.
- Scope and methods: number of included RCTs, total participants, psychiatric diagnosis, type and duration of intervention, comparator class, risk-of-bias methods used by each SRMA, delivery method, primary, follow-up timing, and publication-bias assessment.
- Outcomes and effects: headline pooled effect metric and direction for the SRMA’s primary outcome, statistical significance at post-treatment, and any prespecified subgroup/sensitivity findings.
- Equity descriptors (only descriptive): if available at review-level, PROGRESS-Plus variables, such as age distribution, sex, gender, ethnicity categories, and other vulnerable groups.

All data will be extracted from published documents only; hence, authors of the included SRMAs will not be contacted for additional information. We will not compute or pool effect sizes, re-extract study-level (RCT-per-RCT) participant characteristics, or perform any new meta-analysis.

For the narrative synthesis, we will report whether SRMAs indicate that the included RCTs:

- distinguish drug-free/drug-naïve participants from those receiving concurrent pharmacotherapy;
- enrolled psychotherapy-naïve participants or participants with prior exposure to other modalities, since prior therapy may influence treatment outcomes and differ systematically between CBT and PPT trials;
- specify how treatment duration was classified (e.g., short-term versus long-term PPT; brief versus extended CBT) and whether subgroup/sensitivity analyses were conducted by follow-up length;
- restricted outcomes to symptom-reduction questionnaires (e.g., Hamilton Rating Scale for Depression); and

### 2.6 Quality assessment

Each SRMAs will be appraised independently by two reviewers using AMSTAR-2 (17). AMSTAR-2 consists of 16 items, including seven critical domains. AMSTAR-2 assesses six key domains: (1) prior registration of the protocol; (2) comprehensiveness and adequacy of the literature search; (3)relevance of the methods used to combine studies; (4) assessment of the risk-of-bias in the included studies; (5) clarity in the description of the included studies; and (6) consideration of publication bias (17). AMSTAR-2 results are classified into four levels: as “high” (none or one non-critical weakness), “moderate” (more than one non-critical weakness), “low” (one critical flaw with or without non-critical weakness), and “critically low” (more than one critical failure with or without non-critical weakness (17). We will report item-level compliance, overall categories (counts and proportions), and an exploratory continuous score (labeled as such, because AMSTAR-2 does not endorse numeric scoring as the primary result).

To evaluate the certainty of the evidence, each pair of reviewers will independently apply GRADE approach for SRMA’s stated primary outcomes (SRMA x Outcome). We will assign one GRADE rating per SRMA for each outcome at the prioritized time point, using only information reported in the SRMA (we will not re-extract trial-level data or re-meta-analyze). Since SRMAs usually report a variety of outcomes and time points, we will use a pre-specified hierarchy to ensure comparability:

1. Primary outcome: the SRMA’s pre-specified primary symptom outcome at acute post-treatment.
2. If a primary outcome is not declared: the most used validated symptom measure for the target diagnosis in that SRMA.
3. Follow-up outcomes (short-, medium-, long-term as defined by each SRMA) will be summarized in Supplementary Material and used in sensitivity summaries.

GRADE classifies certainty of evidence into four levels: “high”, “moderate”, “low”, and “very low”, considering GRADE’s five downgrading domains (risk of bias, publication bias, imprecision, inconsistency, and indirectness) and, where applicable, three upgrading factors (large and consistent effect, dose-response, and residual confounding) (25, 26). Since our eligibility includes RCT-only SRMAs, evidence bodies start at “high” and may be downgraded based on the five standard domains (risk of bias, inconsistency, indirectness, imprecision, and publication bias). Upgrading factors will not be applied, as these apply to observational evidence starting at “low”. Final ratings will be categorized as high, moderate, low, or very low.

For the prioritized outcome in each SRMA, imprecision factor will be judged using the following sequence:

- Minimal clinically important difference (MCID) (27) rule: when a credible MCID exists for the instrument used, we will consider the 95% CI around the SRMA’s pooled effect. If the CI crosses the MCID boundary (i.e., includes both clinically important benefit and no important benefit/harms), we will downgrade for imprecision.
- Optimal information size (OIS) fallback: if no credible MCID is documented, we will judge imprecision using OIS and CI width. Where total information is clearly below an adequate sample for a minimally important effect (per GRADE guidance) and/or the CI spans both benefit and no benefit, we will downgrade.

All imprecision judgments will be explicitly justified in domain-level notes.

For each prioritized outcome, we will compile Summary of Findings (SoF) tables by modality (CBT, PPT) that list: outcome, effect metric (as reported), time point, total participants/RCTs, and GRADE certainty with domain-level notes. Two reviewers will independently rate domains; κ statistics and adjudication procedures will be reported.

### 2.7 Data synthesis

Data will be synthesized using a mixed approach, integrating both narrative and quantitative approaches. For narrative synthesis and visuals, we will describe patterns in methods and reporting (e.g., protocol registration, comparator types, publication-bias checks) and display: a PRISMA flow diagram; a citation matrix with Corrected Covered Area (CCA) (28), and citation families; and summary graphics (e.g., heatmaps of AMSTAR-2 items by modality; harvest plots showing direction/significance across diagnoses; bubble plots relating AMSTAR-2 and GRADE to number of included RCTs and year). Sensitivity summaries will be presented side-by-side to show robustness. Quantitatively, we will summarize characteristics separately for SRMAs of CBT and PPT. For each modality, we will tabulate: a) bibliometrics (year, journal, language, authors); (b) scope/methods (psychiatric diagnosis, number of included RCTs and total participants, treatment dose/duration, follow-up timing, comparator class, measurement instruments, risk-of-bias and publication-bias tools); (c) methodological appraisals (AMSTAR-2 item compliance and overall rating, GRADE certainty of evidence for primary outcome); (d) results as reported by each SRMA (outcome measures, effect sizes, direction and statistical significance at post-treatment and, if available, follow-up). We will not harmonize or pool effects across reviews. All results will be presented exactly as reported by the SRMAs.

### 2.8 Data analysis

Analyses will be performed in JASP^®^ (Jeffreys’s Amazing Statistics Program). We will proceed in five steps:

1. Descriptive statistics (specific objectives: 1 to 3): Means, standard deviations, medians and interquartile ranges (IQRs) will be separately calculated by modality (CBT and PPT) for key variables (e.g., number of reviews, AMSTAR-2 scores, GRADE levels, diagnosis group, and comparator class).
2. Reliability (specific objectives: 1 to 3): Cohen’s Kappa (κ) will assess agreement during screening, extraction, AMSTAR-2 and GRADE between reviewers and modalities.
3. Correlation and regression (specific objective: 4): Pearson/Spearman correlations and linear models to explore links in AMSTAR-2 and GRADE to SRMA-level effect patterns; cluster-robust (Huber-White) standard errors with clusters defined by citation families to address dependence due to overlap; leave-one-out (SRMA and family levels) for influence checks.
4. Group comparisons of AMSTAR-2 ratings (specific objective: 5): t-tests or nonparametric equivalents for continuous AMSTAR-2 scores, as dictated by data distribution; χ² or ordinal logistic regression for AMSTAR-2 categories across diagnoses and modalities.
5. Multiple testing: Holm-Bonferroni analysis will be conducted to correct for multiple comparisons.

#### 2.8.1 Study-overlap management

We will construct a citation matrix (SRMAs x RCTs), quantify overlap via Corrected Covered Area approach (27), and identify citation families of SRMAs addressing the same PICO or diagnosis with substantial overlap. Two analysis sets will be reported:

1. All-SRMAs set (primary), including eligible SRMAs; and
2. Best-evidence set (non-overlapping), retaining one SRMA per citation family using the following hierarchy: (i) most recent; (ii) higher AMSTAR-2; and (iii) larger RCT count.

We will report overlap diagnostics (number of families, median and maximum overlap, proportion of unique RCTs per SRMA) and compare results across these sets.

#### 2.8.2 Sensitivity analyses

To test the robustness of descriptive summaries, group comparisons, concordance, and association results, we will conduct the following pre-specified sensitivity analyses:

- Outliers and influence checks: We will conduct a leave-one-out analysis to examine SRMAs with unusually extreme values (outliers) and their influences on correlation and regression findings.
- AMSTAR-2 thresholding: We will re-run analyses after (i) excluding “critically low” SRMAs and (ii) excluding “low + critically low” SRMAs. Then, we will compare effect-pattern stability and summary statistics across thresholds, highlighting any reversals or significant shifts in magnitude or precision.
- Comparator class: As weak comparators can inflate effects relative to active controls, we will repeat analyses including only SRMAs that compare CBT or PPT with active comparators (i.e., other psychotherapies and/or pharmacological comparators). In a further analysis, we will include TAU/waitlist/no-treatment and compare results. We will report any shifts in CBT–PPT differences and certainty of evidence patterns.
- Diagnosis subsets: As cross-diagnosis may mask disorder-specific patterns, we will perform analyses within each diagnostic group (e.g., depression, anxiety, trauma-related, OCD, BPD, etc.), and, upon further analysis, exclude mixed-diagnosis SRMAs. We will compare modality patterns, quality, and certainty of evidence profiles within- versus across-diagnoses patterns.

To all sensitivities analyses, conclusions will be considered robust when the direction of modality (CBT–PPT) comparisons and qualitative inferences about quality or certainty of evidence remain unchanged and quantitative differences stay within the original 95% CIs or reflect only minor precision changes. Divergences will be summarized narratively.

## 3 Expected results, implications, and limitations

This umbrella review aims to contribute to ongoing discussions regarding the effectiveness of psychotherapy by analyzing review-level methodological quality and certainty of evidence of SRMAs in two of the most used theoretical approaches in the field. Particular attention is given to the ethical implications of psychological treatments and their application across diverse health and social care contexts. It is important to note that this review does not seek to compare the effectiveness of distinct methodological approaches. Therefore, any perceived superiority of one approach over another would be a misinterpretation of our intent. Additionally, we acknowledge that the number of studies included may influence the analyses and outcomes in ways that do not fully reflect reality, especially considering the limitations imposed during the database search. Limitations include language restrictions (potential language bias), reliance on review-level reporting (no trial-level verification), residual double-counting risk despite overlap management, usage of psychology-specific descriptors, and year of publication restrictions. Since this umbrella review depends on the methodological reporting of included SRMAs and will not re-analyze primary trials, we acknowledge that it may inherit SRMAs’ limitations.
